# Effect of exoskeleton-assisted Body Weight-Supported Treadmill Training on gait function for patients with chronic stroke: a scoping review

**DOI:** 10.1186/s12984-022-01111-6

**Published:** 2022-12-21

**Authors:** Rieko Yamamoto, Shun Sasaki, Wataru Kuwahara, Michiyuki Kawakami, Fuminari Kaneko

**Affiliations:** 1 Department of Rehabilitation Medicine, Keio University School of Medicine, 35 Shinanomachi, Shjinjuku, Tokyo, Japan; 2 Department of Artificial Environment, Safety, Environment and System Engineering, Yokohama National University Graduate School of Environment and Information Sciences, 79-7, Tokiwadai, Hodogaya, Yokohama, Japan; 3 Center for Environmental and Health Sciences, Hokkaido University, Kita 12, Nishi 7, Kita-ku, Sapporo, Japan; 4 Division of Health Promotion, ARCE Inc., Sagamihara, Japan; 5 Department of Physical Therapy, Graduate School of Health Sciences, Tokyo Metropolitan University, 7-2-10 Higashi-Oku, Arakawa-ku, Tokyo, Japan

**Keywords:** Robot-assisted gait training, Chronic stroke, Gait exercise, Body Weight-Supported Treadmill Training, Exoskeleton, Scoping review

## Abstract

**Background:**

Therapeutic exercise for gait function using an exoskeleton-assisted Body Weight Supported Treadmill Training (BWSTT) has been identified as a potential intervention that allows for task-based repetitive training with appropriate kinematics while adjusting the amount of body weight support (BWS). Nonetheless, its effect on gait in patients with stroke in the chronic phase are yet to be clarified. The primary aim of this scoping review was to present the status of effectiveness of exoskeleton-assisted BWSTT in patients with chronic stroke. The secondary aims were to summarise intervention protocols, types and functions of BWSTT exoskeletal robotic devices currently used clinically.

**Method and results:**

Articles were accessed and collected from PubMed, Ovid MEDLINE, Cochrane Central Register of Controlled Trials, and Web of Science databases, which were completed in October 2020. Articles were included if the subjects were adults with stroke in the chronic phase (onset ≥ 6 months) and if they utilised a robotic exoskeleton with treadmill and body weight support and investigated the efficacy of gait exercise. A total of 721 studies were identified, of which 11 randomised controlled trials were selected. All included studies were published from 2008 to 2020. Overall, 309 subjects were enrolled; of these, 241 (156 males, 85 females) participated. Walking outcome measures were used more often to evaluate the functional aspects of gait than to evaluate gait independence. In 10 of 11 studies, showed the effectiveness of exoskeleton robot-assisted BWSTT in terms of outcomes contributing to improved gait function. Two studies reported that exoskeleton-assisted BWSTT with combination therapy was significantly more effective in improving than exoskeleton-assisted BWSTT alone. However, no significant difference was identified between the groups; compared with therapist-assisted BWSTT groups, exoskeleton-assisted BWSTT groups did not exhibit significant change.

**Conclusion:**

This review suggests that exoskeleton-assisted BWSTT for patients with chronic stroke may be effective in improving walking function. However, the potential may be “to assist” and not because of using the robot. Further studies are required to verify its efficacy and strengthen evidence on intervention protocols.

## Background

It is estimated that 15 million people worldwide have suffered from a stroke, with approximately 5 million living with permanent disability [1]. Almost 50% of stroke patients are unable to walk after stroke onset, and, even after intensive rehabilitation, 30–40% of these patients still have limited ability to walk [2–4]. Exoskeleton-assisted Body Weight Supported Treadmill Training (BWSTT) has gained attention over the past few decades as a popular method of post-stroke gait training due to its advantages for task-based repetitive training [5–10]. However, for patients with stroke in the chronic phase, improvements in gait, its outcome measures and specific intervention protocols have yet to be clarified.

### Chronic stroke

In this review, we defined chronic phase in stroke as equal to or more than 6 months after stroke onset. It has been suggested that the majority of functional recovery after stroke onset occurs in the acute phase and plateaus from 3 to 6 months after onset. However, previous studies have shown that specialised and intensive training can improve motor function in patients with chronic stroke who have motor dysfunction [11–14]. Moreover, the degree and amount of improvement in motor function has been reported to be correlated with the intensity and frequency of rehabilitation [15–17].

### Robotic devices in gait rehabilitation

**Fig. 1:**
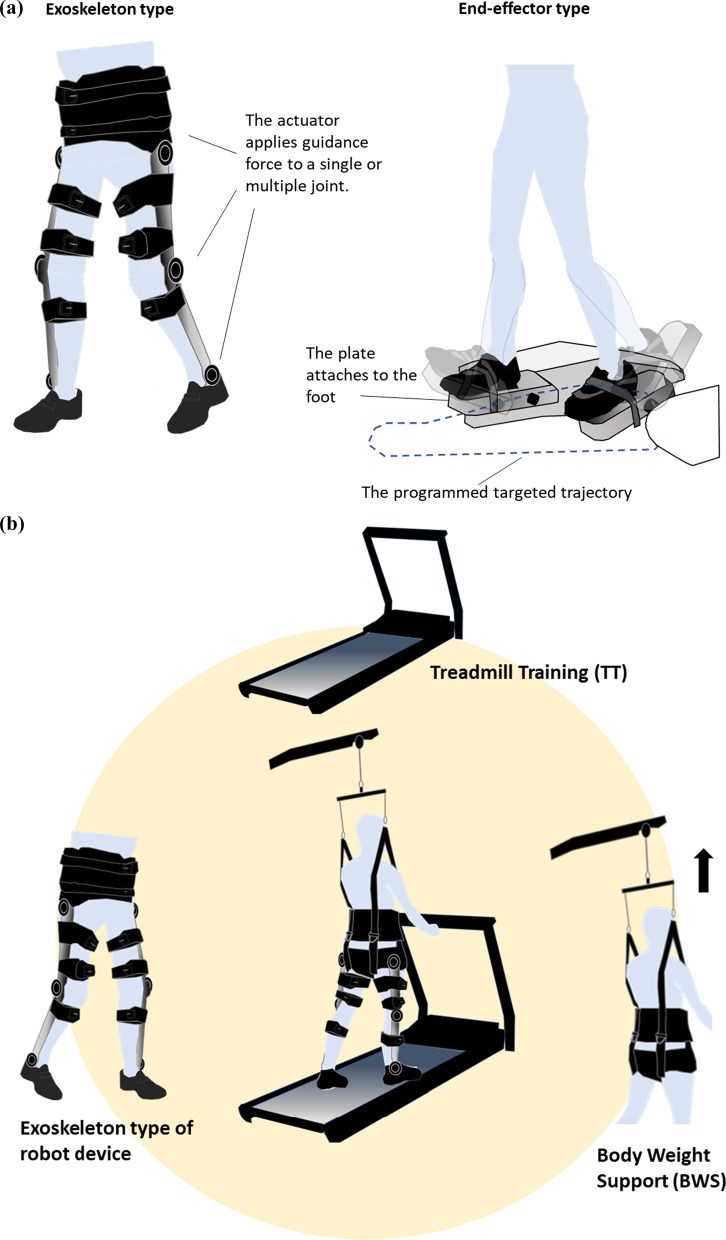
**a** Major types of robotic devices for robot-assisted therapeutic gait rehabilitation. The figure shows the Exoskeleton type (left) and the End-effector type (right). **b** Exoskeleton type of robot device with Body Weight Supported Treadmill Training (BWSTT)

A search in the PubMed for articles related to gait-assist robots used in rehabilitation yielded no results prior to 1989, although the number of articles rapidly increased from 2003 to 2020 as the field was recognised in rehabilitation. The application of robotic technology to rehabilitation has substantially increased in recent years [18–22] and several gait-assistive robotic devices are already available on the market [5–10, 23, 24]. The available assistive robot systems include HAL (CYBERDYNE Inc., Japan), Welwalk (Toyota Motor Corporation, Japan), Lokomat (Hocoma AG, Switzerland), Ekso (Ekso Bionics, USA), and many others are currently undergoing development. All these devices possess one or more of the following functions: a body-weight-support device, a treadmill, or an overground walking system. The devices used for gait exercises utilise electromechanically actuated motors that control movement and exert force on the joints or parts of the lower limbs. These are categorised as exoskeleton type, end-effector type and other [25] (Table 1, Fig. 1a, b). The exoskeleton type is wearable and assists the patient by applying output torque directly at the targeted lower limb joints during gait training. The timing and intensity of the assistance is programmed and given during the entire gait cycle or in a specific phase. The end-effector type is a device placed on the plantar that provides assistance of the ideal gait trajectory to the foot (peripheral). Types stated as other included powered walking frames, powered ankle foot orthosis or non-exoskeleton wire-driven types. Unlike end-effector type robots, which have a fixed end and input programmed motion trajectory, exoskeletons are intended to compensate for lost gait function, characterised by dynamic assistance or control of the rotational motion of the target joints. In equipment associated with exoskeletons (Fig. 1b), the adjustable body weight support (BWS) function, which prevents falls, may safely accommodate patients with a wide range of gait function levels from Functional Ambulation Categories (FAC) 0–4 [26]. The treadmill also facilitates speed adjustment and repetitive gait input in a set position. BWSTT has been reported to significantly improve balance, gait speed, and endurance in stroke patients [27, 28]. Furthermore, it has been reported that gait training with adjusted weight bearing instead of full weight improves walking speed and endurance on level ground, leading to improved gait [29, 30]. BWSTT is highly effective in improving gait in patients who have suffered subacute stroke, but its effectiveness is not clear in chronic stroke [27, 29, 31]. It has been reported that gait-assistive robot training has an impact on gait improvement [27]. On the other hand, literature comparing the effects in terms of differences by type of gait-assist robot and stage of the target patients is limited, and effects on specific endpoints are not yet clear [25, 32, 33]. Moreover, results may differ depending on the clinical trial design and intervention protocols.

**Table 1:** Types of robotic devices for therapeutic gait rehabilitation

Type of device		BWS device	Treadmill	Examples of some representative product
Exoskeleton	BWSTT Exoskeleton	Yes	Yes	Lokomat (Hocoma AG, Switzerland), Welwalk (Toyota Motor Corporation, Japan), Walkbot (P&S Mechanics Co. Ltd., Korea)
	Overground Exoskeleton	No	No	HAL(Cyberdyne, Japan), Ekso-GT(Ekso Bionics, USA)
End-effector		Yes	N/A	G-EO System (Reha Technology, Swissland), LokoHelp(Woodway, USA)
Other		–	–	Powered AFO, Walking aids with electric assist functions

*BWSTT* Body Weight Supported and Treadmill Training, *BWS* Body Weight Support

### Gait exercise for stroke

Currently, several forms of intervention in gait training have been proposed for the management of stroke. Conventional physiotherapy for walking after stroke generally includes muscle strengthening, functional task practise, symmetrical movement practise (including weight bearing and shifting training), stepping and single-leg standing targets for practising specific gait phases, circuit training, and neurodevelopmental techniques [34]. Most conventional gait retraining is undertaken with hands-on assistance, which is potentially physically taxing for the therapist.

Frequent intense gait training interventions have been shown to result in higher overall functional improvements in patients with stroke in chronic phase [5–10]. However, the physical burden and time/cost required to maintain such functions are a key challenge amongst therapists, as well as patients in the chronic phase after stroke.

The use of technology-enhanced gait training for rehabilitation which gives mechanically assisted task-based repetitive training is expanding; nonetheless, its competence is still being argued. A previous study reported that individuals who received electromechanical-assisted gait training in combination with physiotherapy after stroke were more likely to achieve independent walking than people who received gait training without these devices [25, 31]. In another study, therapist-assisted locomotor training was superior to robotic-assisted locomotor training amongst ambulatory survivors with chronic stroke [35]. In addition to refining the application of devices and determining patients who may benefit from robot-assisted training, the identification of an effective combination therapy crossover is essential.

In summary, although significant research has been done on exoskeleton robotic rehabilitation, only minimal research has been conducted on its application in patients with chronic stroke. Moreover, investigations on the efficacy of devices used in robotic gait exercise in the chronic phase are limited, and the current situation is unclear. At the same time, while various types of robotic assistive devices have been developed to date, the trends in equipment, design, and functional requirements specifically for the chronic phase are still not well known [27, 29, 31].

However, recent randomised control trials (RCT) focusing on the efficacy of exoskeleton-assisted BWSTT (Fig 1b) for chronic stroke have reported positive improvements in gait [35–45], indicating that there is a potential value in further research to clarify this finding.

Therefore, this study aimed to review and describe the effectiveness of exoskeleton assisted BWSTT in patients with chronic stroke. And the second objectives were to summarise intervention protocols and the types and functions of BWSTT exoskeletal robotic devices currently used clinically.

## Methods

The literature review protocol was developed in accordance with the Preferred Reporting Items for Systematic Reviews and Meta-Analyses (PRISMA) Statement [46], and with reference to the work conducted by Arksey and O'Malley [47], Ferrari [48] and Peters [49].

Articles were accessed and collected from PubMed, Ovid MEDLINE, Cochrane Central Register of Controlled Trials, and Web of Science databases, which were completed on October 27, 2020.

The primary search was conducted using combined terms: (robot* OR exoskeleton OR “powered gait orthosis” OR PGO OR HAL OR “hybrid assistive limb” OR ReWalk OR Ekso OR Indego OR lower-extremity robot OR robotic-assisted OR electromechanical OR mechanically assisted OR powered assisted OR robotic device OR Welwalk OR electromechanical-assisted OR robotic OR end-effector OR assist robot OR GEAR OR robotic orthosis OR rehabilitation robotics OR orthotic devices OR Lokomat) AND (stroke OR post-stroke OR CVA OR “cerebrovascular accident” OR “cerebral infarct” OR “cerebral haemorrhage” OR hemiplegia OR hemiparesis) AND (gait OR walk OR walking OR ambulation OR gait training) AND (chronic OR community OR at home). Additionally, the following parameters were used: clinical trial/ RCT and scientific articles written in English, with its full text available to all the authors. Date was not restricted. Additional references were also identified by manual search, and duplicates were removed.

The inclusion criteria were as follows:

- Interventions, allocated subject group, and outcome measures that refer to the efficacy and/or effectiveness of gait exercise and exoskeleton-assisted BWSTT
- Human subjects: post-stroke, hemiplegia in the chronic phase with onset at ≥ 6 months
- Type of electromechanical robotic exoskeleton used in gait exercise that facilitates movement or exerts force on the hip, knee, or ankle joints

The exclusion criteria were as follows:

- The stated devices were not for use on the lower limbs (upper limbs, hand robots, devices for controlling pelvic motion, etc.)
- The disease stage was not clearly stated or there were stages other than chronic in the target group
- Included healthy participants or children aged < 18 years
- Included participants with mixed diagnosis
- Used only general braces such as ankle foot orthosis and had a drive source that was not controllable electro mechanically
- Used only the following interventions for the subject group: BWS device on a treadmill, electromyographically driven neuromuscular electrical stimulation, and virtual reality (VR)/augmented reality
- Did not apply force on the limb nor was movement facilitated by the device
- Used the end-effector type of device or exoskeleton for overground walking
- Reported only technology development

**Fig. 2:**
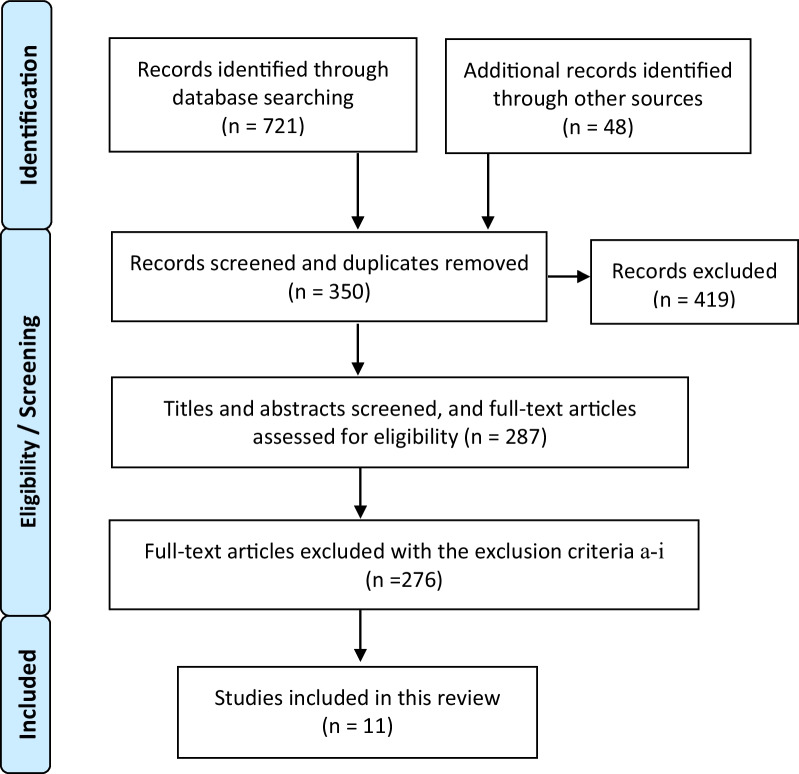
A flow diagram of the selection process. The PRISMA flow diagram [46] of the process of the database searches, the number of abstracts and full texts screened assessed

The selection process is shown in the flow diagram in Fig. 2 [46].

With respect to the reference selection process and the inclusion and exclusion criteria above, the titles and abstracts of potential articles were screened by two reviewers to remove irrelevant studies. Potentially eligible studies were chosen from the remainder if full texts were available.

From the final selected studies, data on the study design and subjects, equipment used for the interventions, gait exercise treatment protocol, evaluation tools, and reporting of information on the effectiveness of clinical gait exercise were extracted from the selected articles to provide information on the results and effects that would be useful in clinical practise (Table 2). Additionally, data on the characteristics, type and functional requirements of the gait assistive robots as devices used in the selected literature were summarised to analyse them for use in the chronic stage (Table 3).

**Table 2:** Literature summary

Author, year Study design	Participants	Participants' walking ability at baseline	Training period and protocol (duration of the intervention, session)	Apparatus and its setting	Outcome
Hornby et al. (2008) [35] RCT	**48** patients with chronic stroke **IG**: robotic LT group (**n = 24**) **CG**: therapist-assisted LT group (**n = 24**)	> 10 min overground without assistance at WS ≤ 0.8 m/s at SSV, using assistive device	**IG:** with guided symmetrical locomotor assistance by robot **CG:** with guided symmetrical locomotor assistance by therapist manually **12 sessions** **30 min** per session	Device: **Lokomat** BWS: weight support% is **25 ± 6.7%** for robotic- assisted and **21 ± 7.5%** for therapist-assisted WS: **up to 3.0 kmph** decreased throughout training	Outcome measures: **WS (SSV and FV), 6MD at SSV, mEFAP, BBS, SF-36, MMT, MAS, CES-D** Result: In therapist-assisted LT, greater improvements in speed and single limb stance time on the impaired leg compared with those in the robotic LT group were found
Belas Dos Santos et al. (2018) [36] RCT	**15** patients with chronic stroke **IG: n = 7** **CG: n = 8**	Baseline and outcome evaluation: BBS, FIM, TUG	**IG:** 2 sessions of PT and 1 session of RAGT **CG:** 2 sessions of PT and 1 session of TAGT **60 min** per session **5-month** protocol	Device: **Lokomat** BWS: gradually **reduced from 50%** of patient weight at the start of the protocol treatment until **a minimum of 10%** at the end WS: low speed between **0.8 kph and 1.5 kph**	Outcome measures: **BBS, TUG, FIM, SARA** Functional scale scores mean values differed significantly between groups. No significant difference for the between-group comparison at both baseline and after treatment
Seo et al (2018) [37] RCT	**12** patients with chronic stroke **IG: n = 6** **CG: n = 6**	Able to walk at least 10 m independently Asymmetrical gait with a step length asymmetry ratio > 1.1 FAC Group 1: 3.3 ± 0 Group 2: 3 3.7 ± 0.2	**IG:** assist-as-needed RAGT for the unaffected limb and fully assisted RAGT for the affected limb **CG:** assist-as-needed RAGT for the affected limb and fully assisted RAGT for the unaffected limb **20 sessions** **2 times** per week **45 min** per session	Device: **Walkbot** BWS: bodyweight support was reduced as the subject's function improved (no clear description present) WS: the treadmill speed was increased to a **maximum of 2.2 km/h** as the subject's function improved	Outcome measures: **NIHSS, FMLE, FAC, MI, and the TCT** Assessed at the baseline (T0) and after 10 (T1) and 20 (T2) training sessions Result: In IG, FMLE, FAC, and MI scores significantly improved at T2 compared to T0. The unaffected limb's step length asymmetry ratio and hip maximal extension moment significantly improved In CG, only FMLE score improved significantly at T2 compared to T0 No significant differences in the between groups analysis was found
Lewek et al. (2009) [38] RCT	**19** participants with chronic stroke **IG:** therapist-assisted LT (**n = 9**) **CG:** robotic-assisted LT (**n = 10**)	Able to walk at least 10 min overground without assistance and at SSV of 0.8 m/s	**IG:** therapist-assisted LT **CG:** robotic-assisted LT **12 sessions** **3 times** per week **60 min** per session	Device: **Lokomat** BWS: **40% BWS** at the first session and reduced as tolerated WS: speed was gradually increased during the first session, **remaining at 3.0 km/h (0.83 m/s)** for the duration of the intervention	Outcome measures: **Maximum joint angles,** **Excursions of the hip, knee, and ankle joints, Hip and knee ACC** Result: Robotic-assisted LT did not demonstrate a significant increase in SSV, hip and knee ACC. Therapist assisted LT resulted in significant improvements in both outcome measures
Westlake et al. (2009) [39] RCT	**16** volunteers with chronic stroke **IG:** Lokomat (**n = 8**) **CG:** manual BWSTT (**n = 8**)	At least unlimited household ambulators (WS > 0.3 m/s)	**IG:** RAGT with Lokomat **CG:** manual BWSTT The fast WS group and slow WS group were assigned to the IG and CG, respectively **12 sessions** **3 times** per week ** ≤ 60 min** per session	Device: **Lokomat** BWS: **35%** at first, reduced in increments of 5% as long as gait quality was maintained WS: maintained **below 0.69 m/s (2.5 km/h)** in the slow groups and **above 0.83 m/s (3 km/h)** in the fast groups	Outcome measures: **SSV, paretic step length ratio, FV, 6MD** Result: IG group, SSV and paretic step length ratio, and four of the six secondary measures improved. Group differences between fast and slow training groups were not stated
Danzl et al. (2013) [40] RCT	**8** subjects with chronic stroke **IG:** active tDCS with RGO **CG:** sham tDCS with RGO	N/A	**IG:** active tDCS with identical locomotor training with a robotic gait orthosis (RGO) **CG:** sham tDCS with identical locomotor training with a robotic gait orthosis (RGO) **12 sessions** **3 times** per week	Device: **Lokomat** BWS: **40–50%** at the beginning of session and then reduced to 20% at the end of protocol training WS: Initially at comfortable speed, followed by progressive, rapid decrease to a speed slow enough to allow subject to initiate movement, thereby increasing engagement with the task and appropriate attentional demands	Outcome measures: **10MWT, BBS, FAC, SIS-16** Result: the active tDCS group showed greater improvement than the sham group in all measures except BBS
Bae et al. (2014) [41] RCT	**20** subjects with chronic stroke **IG:** RAGT with FES (**n = 10**) **CG:** RAGT only (**n = 10**)	Able to walk for 10 m with or without an assistive device	**IG:** robot-assisted gait training and functional electrical stimulation on the ankle dorsiflexor of the affected side **CG:** robot-assisted gait training only **15 sessions** **3 times** per week **30 min** per session with regular **30-min PT** session in both groups	Device: Lokomat BWS: reduced from **40 to 0%** according to the patient’s gait pattern WS: **from 1.2 km/h up to the maximum speed** at which patients could adapt	Outcome measures: **MMAS, TUG, BBS, gait parameters, WS, cadence, step length, stride length, double support, pelvic, hip, knee, ankle joint angle** Result: In IG, Step length and maximal knee extension were significantly greater than those before training and Maximal Knee flexion showed a significant difference between the in IG. The MMAS, BBS, and TUG scores improved significantly after training compared with before training in both groups
Ogino et al (2020) [42] RCT	**19** patients with chronic stroke **IG:** GEAR group (**n = 8**) **CG:** treadmill group (**n = 11**)	All subjects were required to walk on the ground or a treadmill without physical assistance using assistive devices	**IG:** GEAR training **CG:** using only the treadmill function of GEAR **20 sessions** **5 times** per week **60 min** per session Visual and audio feedback was used when needed	Device: **GEAR system (Welwalk)** BWS: - WS: the treadmill speed was **increased from overground maximum speed**. The treadmill speed was **increased by 10%** in the next session, if it was judged safe to do so, and **reduced by 10%** if it was not	Outcome measures: **Abnormal gait pattern, Spatiotemporal gait parameters** Result: In IG, step length and maximal knee extension were significantly greater than those before training Maximal knee flexion showed a significant difference between the experimental and control groups
Ogino et al. (2020) [43]	**19** participants **IG:** GEAR group (**n = 8**) **CG:** treadmill group (**n = 11**)	Independent gait overground without physical assistance, using assistive devices, and bracing below the knee as needed	**IG:** GEAR training **CG:** The treadmill group performed gait training using only the treadmill function of GEAR **20 sessions** **5 times** per week **60 min** per session Visual and audio feedback was used when needed	Device: **GEAR system (Welwalk)** BWS: - WS: the treadmill speed was **increased from overground maximum speed**. The treadmill speed was **increased by 10%** in the next session, if it was judged safe to do so, and **reduced by 10%** if it was not	Outcome measures: **10MWT, TUG, 6MD, SF-8, and GRC scales **Result: In both groups, the MMAS, BBS, and TUG scores showed significant difference before and after training. In IG, WS was significantly increased at completion of training and 1-mo follow-up compared with baseline and GRC scales were significantly increased at completion of training, 1 month follow-up, and 3-month follow-up compared with baseline TUG and 6 min walk were significantly greater in IG than CG at completion of training compared to baseline
Bae et al. (2016) [44] RCT	**34** patients **IG:** HRR-guided high-intensity RAGT group (**n = 17**) **CG:** RPE-guided high-intensity RAGT group (**n = 17**)	Able to walk but with difficulty	**IG:** HRR-guided high-intensity RAGT; RAGT at 70% of HRR **CG:** RPE-guided high-intensity RAGT group; RAGT at RPE of 15 All participants: additional regular 30-min PT **18 sessions** **3 days** per week **30 min** per session,	**Lokomat** BWS: from **40 to 0%** according to individual ability WS: controlled gait speed (**from 1.2 km/h up to**)	Outcome measures: **FMLE, 10MWT, gait parameters** (walking speed, cadence, step length, stride length, swing time, stance time, double, support rate, single support rate, and symmetrical ratio index) Result: The FMLE was significantly higher than that before the intervention in both groups. IG improved significantly more than CG. The value of 10 MWT was significantly higher than that before the intervention in both groups
Erbil et al. (2018) [45] RCT	**IG:** RAT (**n = 29**) **CG:** control (**n = 14**)	Ambulatory patients with or without assistive devices and patients with BBS score ≥ 20 points	**IG:** 30 min of RAT (RoboGait®) plus 60 min of PT **CG:** 90 min of PT **3 weeks during weekdays** In both groups, BoNT‐A injections were applied	Device: **RAT (RoboGait®)** BWS: Body weight support range **0 to approximately** **100 kg (220.5 lbs)**, continuously adjustable without training interruption WS:**0.2–3.2 km/h**	Outcome measures: **MAS and Tardieu Scale, BBS, TUG, and RVGA** Result: In both groups significant improvements were determined regarding spasticity, balance, and gait functions In IG, at post‐treatment Weeks 6 and 12, change from baseline TUG, BBS, RVGA were significantly higher than CG

*BBS* Berg balance scale, *BSW* body weight support, *BWSTT* Body-Weight Supported Treadmill Training, *CG* control group, *FAC* Family Assistance Centre, *FIM* Functional Independence Measure, *FMLE* Functional Mobilisation Lower Extremities, *IG* intervention group, *MMAS* Modified Motor Assessment Scale, *NIHSS* National Institutes of Health Stroke Scale, *PT* Physiotherapy, *SSV* Self-Selected Velocity, *SARA* Scale for Assessment and Rating of Ataxia, *TUG* Timed Up and Go, *RAGT* Robot assisted gait training, *RVGA* Rivermead Visual Gait Assessment, *WS* walking speed

**Table 3:** Description of devices used in the selected literature

Studies and exoskeletons	Design	Type of actuator	Assist joint	Device description
Lokomat [35, 36, 38–41, 44] (Hocoma AG, Switzerland)	Bilateral	Electric Actuation [50] (DC motor)	Hip and knee (ankle passive)	System consisting of a robotic lower-extremity orthosis with: ••••A dynamic body weight support system that supports vertical/lateral centre-of-gravity movement ••••Pelvic position is fixed ••••Adjustable level of assist, gait speed, and guidance force (from 100 to 0%) ••••Computer-regulated motors for individual joints ••••Synchronised treadmill and visual feedback utilities
Walkbot [37] (P&S Mechanics, South Korea)	Bilateral	Electric Actuation [51] (DC brushless motor)	Hip, knee, and ankle	System consisting of a robotic lower-extremity orthosis with: ••••A dynamic body weight support system that supports vertical centre-of-gravity movement ••••Pelvic position is fixed ••••Synchronisation of the hip, knee, and ankle to assist patients in learning correct gait patterns, impedance control: patient's voluntary efforts are detected and patients are allowed to influence gait patterns during rehabilitation, automatic adjustment of leg length, motion analysis: kinetic and kinematic data reconstructed as a 3D image ••••Synchronised treadmill
RoboGait [45] (Bama Technology, Turkey)	Bilateral	Electric Actuation [52] (Linear motor)	Hip and knee	System consisting of a robotic lower-extremity orthosis with: ••••Body weight supported system and pelvic position is fixed ••••Eight force sensors measuring in pairs in each joint and four force sensor amplifiers ••••Synchronised treadmill and biofeedback utilities
GEAR system/Welwalk [42, 43] (Toyota Motor Corporation, Japan)	Unilateral	Electric Actuation [53]	Knee	System consisting of a robotic lower-extremity orthosis with: ••••Body weight supported system ••••Gait phase is calculated using data from the pressure sensor ••••Adjustable level of assist, gait speed, and guidance force (from 100 to 0%) ••••Real-time feedback system for gait characteristics on the monitor screen ••••Recording of quantitative data during intervention with biofeedback system

## Results

The PRISMA flow diagram for this study is presented in Fig. 2. Overall, 721 studies were identified through database search, and 48 additional potentially relevant studies were found through manual search. From a total of 769 studies, 350 were retrieved by screening the studies’ written language and article type. Additionally, duplicate records were removed. After assessing the eligibility of articles based on the title, abstract, and full text, 11 were selected. All included studies were published from 2008 to 2020. Six studies were published between 2015 and 2020 and five studies were published before 2014.

### Study design

All included studies were RCTs or randomised crossover trials. Most studies included fewer than 30 participants and were recognised as small-scale pilot studies except for three RCTs [35, 44, 45] which included 34, 48, and 48 participants.

### Participants

A total of 309 subjects were enrolled in all included studies; of these, 241 (156 males, 85 females) participated. All participants were patients with chronic stroke with onset at ≥ 6 months. The mean age was 57.8 ± 7.0 years. The subjects were community residents or recruited from the outpatient department in three studies [42, 43, 45], were hospitalised in four studies [36, 37, 41, 44], and belonged to other categories or were unknown in four studies [35, 38–40].

In addition, in a few studies, the subjects were conditioned to a certain level of walking prior to the intervention (a level of independence in which the subjects were able to walk for more than 10 m without a walking assistive device or without receiving walking assistance). Few studies also involved the use of a cane, orthotic device, or walker to walk for more than 10 m.

Three reports specified a walking speed of 0.3 m/s [39] and 0.8 m/s or more [35, 38]. Furthermore, four studies used the Berg Balance Scale (BBS) [36, 45], Timed Up and Go Test [36], Functional Independence Measure [36], Modified Ashworth Scale [42, 43], etc. at the functional level.

### Training period

The duration of intervention and the duration of a session differed in each study. In the included studies, the duration of intervention ranged from 3 weeks [45] to 5 months [36], and the number of training sessions varied from nine [45] to 12 sessions [35, 38–40], 18 sessions [44], and 20 sessions [36, 41, 42].

The duration of one session of lower-limb robotic gait training was 30 min [38, 39, 41, 44, 45], 45 min [37], and 60 min [36, 42, 43], with 30 min being set in approximately half of the studies. In addition, the frequency range was, in descending order of days, five days a week [43, 43], three days a week [38–41, 44, 45], and two days a week [41]. Most of the studies were conducted five or three times a week.

### Training protocol and subject group characteristics

All selected studies utilised gait training on a treadmill and included BWSTT robot-assisted gait training as the intervention. The characteristics of the training protocol and comparison groups were as follows: the most frequent comparisons were robot-assisted gait training groups vs. therapist-assisted groups [35, 36, 38, 39], and the second most frequent comparisons were robot-assisted gait training with combination therapy groups vs. robot-assisted gait training groups. Combination therapy was described as transcranial direct current stimulation (tDCS) [40] or functional electrical stimulation (FES) [41]. Robot-assisted gait training with conventional therapy was compared with robot-assisted gait training alone in one study [45], the effects of robot-assisted gait training were compared between the affected and non-affected sides in another study [37], and the intervention was evaluated in BWSTT robot-assisted gait training groups vs. a robot-free group in two studies [42, 43].

Additional studies compared an exercise loading index, heart rate reserve, and rating of perceived exertion for robot-assisted gait training [44] and compared the effects of different walking speeds between robot-assisted groups [54].

### Outcome measures

Walking outcome measures were used more often to evaluate the functional aspects of gait than to evaluate gait independence. Two studies evaluated the Functional Ambulation Category [37, 40] and Rivermead Visual Gait Assessment [45] as the measures of gait and mobility independence. Two studies either measured the walking speed or utilised the 10-m walk test as a quantitative assessment of gait function [40, 44]. In addition, two studies [35, 43] assessed gait endurance using the 6-min walk test, whereas four studies [36, 39, 40, 43] assessed it using the Timed Up and Go Test. Four studies [36, 40–42] assessed balance ability using the BBS. Three studies evaluated spatiotemporal parameters such as stance time and stride length, floor reaction force data, and kinematic gait parameters such as angular changes in each joint were measured using a 3D motion capture system [38, 42, 44].

Except for those mentioned above, with respect to performance-based outcome measures that are directly related to gait assessment, the Modified Ashworth Scale [45] was investigated as a score of spasticity and a scale of sensorimotor function in stroke. In another study using combination therapy, robot-assisted gait training was undertaken with neurophysiological assessment using an electroencephalogram (EEG). Furthermore, four of the 11 studies included self-reported assessments for depression and satisfaction with treatment (Hamilton Rating Scale for Depression, Center for Epidemiological Studies-Depression Scale [35, 36], Global Rating of Change [42]).

### Types of exoskeletons and their control methods

In all selected studies, exoskeletons utilised a combination of a treadmill and BWS system. In this review, the most utilised exoskeleton was Lokomat, which was used in seven studies [35, 36, 38–41, 44]. The Wellwalk prototype was used in two studies. [42, 43]. Other exoskeletons used were Walkbot [37] and RoboGait [45]. Wellwalk was the only unilateral type; the other exoskeletons had a bilateral set-up.

Lower-limb robots utilised the following joint assistance: Walkbot: hip knee and ankle tri-joint control [37]; Lokomat and RoboGait: hip and knee bi-joint control [35]; and Gait Exercise Assist Robot (GEAR) system/Wellwalk: knee and ankle joint bi-joint control [42, 43]. These four lower-limb robotic devices have an integrated BWS system and treadmill. Of these, Lokomat [35, 36, 38–41, 44], Walkbot [37], and RoboGait [45] have a pelvic immobilisation device in addition to the assisted joint to fix the trajectory of the centre-of-gravity movement in the pelvis. In the GEAR system/Welwalk [42, 43], the pelvic movement is not fixed and is relatively free. All the above-mentioned devices are equipped with visual and auditory feedback functions and the ability to see the patient’s gait.

Table 3 provides further details about all four devices, including their design and type of actuator, and describes these devices, including control strategies and their function.

### Walking speed and BWS in treadmill gait training

The setting criteria for the treadmill walking speed varied. Some studies did not state a numerical speed but specified it as the maximum speed that the patients could achieve [42, 43]. Alternatively, some studies used an individual patient’s comfortable speed that decreased to the appropriate speed [40] or set a constant speed (2.5 km/h to 3.0 km/h [39], 0.2 km/h to 3.2 km/h [45]). In addition, some had a fixed starting speed, with gradual increases in speed in accordance with the patients’ maximal effort and improvement (starting at 0.8 km/h to 1.5 km/h [36] or 1.2 km/h [41, 44]). Others had a fixed maximum speed, and the speed gradually increased within the upper limit of its maximum value (up to 2.2 km/h [37] and up to 3.0 km/h [35, 38]).

The criteria for BWS also varied widely. In some studies, BWS at the beginning of the protocol was set as the percentage of each patient’s body weight and was gradually decreased based on the patient’s ability or improvement [36, 38, 40, 41, 44]. In particular, most studies were based on a gradual decrease in the upper limit of BWS from 40% [38, 40, 41, 44] and from 50% [36] at the start of the protocol. The lower limit was set in the range of 0–20%, depending on the patient’s improvement and change in ability [36, 40, 41, 44].

### Efficacy of BWSTT exoskeleton-assisted gait training and results of individual studies

In all 11 RCTs, the effects of exoskeletal robot-assisted training varied due to different intervention protocols, intervention periods, and lower-limb robotic devices used (Table 2). The results were classified according to the characteristics of the subject groups as follows:

Four studies [35, 36, 38, 39] were categorised into BWSTT-robot assisted gait training (BWSTT-RAGT) vs BWSTT- therapist assisted gait training (BWSTT- TAGT) (Table 4, 4.1). In all three studies [35, 36, 39], both groups showed improvement in gait outcome measures (Table 4) when comparing within each group. However, no significant between-group difference was observed [35, 36, 39]. In one study [35], the BWSTT-RAGT group had a lower improvement in walking speed (self-selected velocity and fast velocity) than the BWSTT-TAGT group. In addition, the results of one study [38] indicated that robot-assisted gait training groups did not show a significant change within and between groups.

**Table 4:** Summary of efficacy of BWSTT exoskeleton-assisted gait training

4.1 BSWTT-RAGT vs BSWTT-TAGT					
Author		Hornby et al. [35]	Belas Dos Santos et al. [36]	Lewek et al. [38]	Westlake et al. [39]
Additional treatment provided		N/A	Conventional PT	N/A	N/A
Results in BSWTT-RAGT groups (pre, post change, p < 0.05)	Body function/structure level	**SSV** + 0.07, d = 0.29 **FV** + 0.06, d = 0.19	**SARA** − 3.5, d = 0.49 **BBS** + 5.8, d = 0.31 **TUG** − 0:19 s, d = 0.64	No change	**FMLE** + 2.6, d = 0.56, **BBS** + 1.4, d = 0.2 **SS** + 0.01 m/s, d = 0.29 **FV** + 0.09 m/s, d = 0.15 **SLR (abs)** − 0.16, d = 0.31
	Activity level	–	**FIM** + 4.6, d = 0.34	–	–
Results between groups (p < 0.05)		BWSTT-TAGT group showed greater improvements in **SSV** + 0.06 m/s, d = 0.65, **FV** + 0.07 m/s, d = 0.69, **Single limb stance time at FV**: + 2.4 ± 3.7%, d = 0.91	No significant difference	No significant difference	No significant difference

4.2 BSWTT-RAGT vs BSWTT-RAGT with combination therapy			
Author		Danzl et al. [40]	Bae et al. [41]
Additional treatment provided		tDCS for experimental group	FES for experimental group Conventional PT
Results in BSWTT-RAGT groups (pre, post change, p < 0.05)	Body function/structure level	**10MWT** + improved	**MAS** + 1.92, d = 0.27 **TUG** − 5.63 s, d = 0.38 **BBS** + 3.43, d = 0.41 **Gait speed** + 0.007 m/s, d = 0.47 **Step length** + 0.05, d = 0.43 **Stride length** + 0.33, d = 0.33 **Maximal Knee flexion** + 18.747 d = 1.07 **Maximal Knee flexion** + 6.904 d = 0.58
	Activity level	**FAC** + improved **SIS-16** + improved	–
Results between groups (p < 0.05)		BSWTT-RAGT with active tDCS group showed greater improvement than the sham group in **10MWT, FAC**, and **SIS-16** measures except BBS	BSWTT-RAGT with FES group showed a significantly greater in **Maximal Knee flexion** + 8.97, d = 0.56

4.3 BSWTT-RAGT vs BWSTT			
Author		Ogino et al. [42]	Ogino et al. [43]
Additional treatment provided		N/A	N/A
Results in BSWTT-RAGT groups (pre, post change, p < 0.05)	Body function/structure level	**GRC scale (change of gait)** + improved	**10MWT** + 0.09 m/s
	Activity level	–	–
Results between groups (p < 0.05)		No significant difference	BSWTT-RAGT group were significantly improved in **TUG** (r = 0.57), **6-min walk** (r = 0.51) and **score of general health in SF-8** (r = 0.49)

4.4 Other				
Author		Assist unaffected limb vs affected limb	HRR vs RPE guided BSWTT-RAGT	BSWTT-RAGT vs Conventional PT
		Seo et al. [37]	Bae et al. [44]	Erbil et al. [45]
Additional treatment provided		N/A	N/A	Conventional PT BoNT-A
Results in BSWTT-RAGT groups (pre, post change, p < 0.05)	Body function/structure level	Assist US: **FMLE** + 3.2, d = 1.18 **MI** + 11.7, d = 2.32 **Step length asymmetry ratio** -0.2, d = 2.0 **Hip maximal extension moment (US)** -0.5, d = 1.79 Assist AS: **FMLE** + 2.7, d = 1.29 **Ankle maximal dorsiflexion angle (US)** -8.9, d = 3.26	HRR guided: **FMLE** + 3.67, d = 0.23, **10MWT** + 0.22 m/s, d = 0.80, **WS** + 0.20 m/s, d = 1.53 And Improved in **Stride length, Cadence**, **Single and Double support rate, Swing time,** S**tance time, Step length,** and **Symmetrical index** RPE guided: **FMLE** + 2.20, d = 0.63, **10MWT** + 0.13 m/s, d = 0.41, **WS** + 0.14 m/s, d = 0.14 And Improved in **Stride length, Cadence**, **Single support rate Single and Double support rate, Swing time**, **Stance time**, **Step length**, **Symmetrical index**	**MAS** − 1.5, d = 2.94 **Tardieu Scale** (spasticity grade) − 0.2, d = 0.44 **BBS** + 2.7, d = 0.29 **TUG** + 5.7, d = 0.66
	Activity level	Assist US: **FAC** + 0.7, d = 2.33	–	**RVGA** + 5.3, d = 1.0
Results between groups (p < 0.05)		No significant difference	HRR-guided group showed significantly improved in compared to RPE-guided group in **FMLE, 10MWT, WS**, **Stride length**, **Cadence**, **Single support rate**, **Single and Double support rate**, **Swing time**, **Symmetrical index**	BSWTT-RAGT group is significantly higher in TUG, BBS, and RVGA

Each study was categorised according to the characteristics of the comparison group under investigation. The results of BWSTT-RAGT intervention groups in pre-post change (p < 0.05) and results compared to the control group are shown. Descriptive values are presented as the mean change and d describes effect size. Results were categorised as Body function/structure level and Activity level [55, 56]. *RAGT* Robot-assisted gait training, *TAGT* Therapist-assisted gait training, *BWSTT* Body-Weight Supported Treadmill Training, *PT* Physiotherapy, *AS* Affected side, *US* Unaffected side, *BBS* Berg balance scale, *BWS* body weight support, *FAC* Family Assistance Centre, *FIM* Functional Independence Measure, FMLE, Functional Mobilisation Lower Extremities; *MMAS* Modified Motor Assessment Scale, *SARA* Scale for Assessment and Rating of Ataxia, *TUG* Timed Up and Go, *RVGA* Rivermead Visual Gait Assessment, *WS* walking speed, *FES* functional electrical stimulation, *GRC scale* Global rating of change scale

In BWSTT-RAGT vs BWSTT-RAGT with combination therapy (Table 4, 4.2), two studies [40, 41] reported that robot-assisted BWSTT-RAGT with combination therapy was significantly more effective in improving gait mainly in activity level of outcome within and between groups than BWSTT-RAGT alone. Furthermore, in a study using tDCS as combination therapy [40], the BWSTT-RAGT with active tDCS group showed greater improvements in 10 MWT (10 Metre Walk Test), FAC, and SIS-16 (Stroke Impact Scale-16) measures except for the BBS than the sham group. In another trial investigating the effects of an intervention combining robot-assisted gait training and FES, maximal knee flexion during gait was significantly greater than that before training in the BWSTT-RAGT with FES group [41].

Table 4, 4.3 shows studies comparing BWSTT-RAGT and BWSTT, two of which were applicable [42, 43]. In these two studies, effects were found within the BWSTT-RAGT group for activity level measures, including the 10MWT. There were no significant differences between the groups regarding kinetics and gait pattern changes [42]. However, quantitative measures of gait, such as TUG and 6-min walk, in the BWSTT-RAGT were higher than in the BWSTT group [43].

In other categories summarised in Table 4, 4.4, one study [45] which compared with conventional physiotherapy showed greater improvement in gait function between group in BSWTT-RAGT group. One of the studies compared the effect of robot assistance on the unaffected limb or affected limb during BWSTT-RAGT [37], and others compared the method of guiding the target of BWSTT-RAGT [38]. Both studies [37, 44] showed significant improvement in outcome measures both in body function/structure and activity level in both conducted BWSTT-RAGT groups.

## Discussion

The purpose of this review was to present and assess the status of effectiveness of robot-assisted BWSTT. Eleven studies were included, indicating that only a small number of RCTs on this topic have been published. As the date of publishing ranged from 2008 to 2020, it could be said that this is a relatively new field. Additionally, more positive improvements in walking in the acute to subacute phase have been reported [26, 31, 57]. There exist few reviews about BWSTT robot-assisted gait exercise that focus on the chronic phase after stroke; hence, its efficacy remains unclarified.

### Effect of robot-assisted gait training in the chronic phase after stroke

There is an expectation that robotic rehabilitation will lead to a paradigm shift in work due to the therapeutic effect on the patient and the reduced burden on the therapist. From this perspective, the results of this review lead to the conclusion that it is not possible to conclude that BWSTT-RAGT is significantly more effective.

We have identified from the study protocol that it is relevant to address papers that present the results of the target intervention group which investigate the effect of exoskeleton used RAGT from the perspective of scoping the current RAGT.

Within the BWSTT-RAGT group, pre- and post-intervention results demonstrated that 10 out of 11 studies [35–37, 39–45] showed a significant improvement in some gait function outcome. Furthermore, there was no significant worsening of gait function in all selected studies [35–45].

The four of the 11 selected studies compared BWSTT-RAGT with BWSTT-TAGT, indicated that there was either no significant difference between the groups [36, 38, 39] or a predominant change in the therapist-assisted group as compared to that in the group receiving conventional gait exercise and the group receiving robot-assisted gait exercise [35]. Therefore, we did not reach the conclusion that the robot was more effective than the therapist for chronic stroke patients.

On other hand, those comparing the BWSTT-RAGT only group to the BWSTT-RAGT with combination therapy group (tDCS [40], FES [41]) have reported significant effects on improving gait outcome measures in between groups. Further research is encouraged, as BWSTT-RAGT with combination therapy may further enhance the efficacy of BWSTT-RAGT. Furthermore, there are studies excluded from the conditions for acceptance, although the following studies have been reported. In studies reporting on improvements in brain function levels that may be involved in the improvement of gait function and an RCT focusing on robot assisted gait training with combination therapy using visual stimulation with VR had shown and identified three main areas of brain activity, as measured by electroencephalography that were significantly evident in the robot assist with VR group [58]. Another RCT [59] of BSWTT-RAGT reported improvements in cognitive flexibility and shifting skills, selective attention/visual research, and quality of life.

The results of two out of 11 references [42, 43], which were from the same research group, and BWSTT-RAGT was effective between the groups, depending on the outcome. These indicate that BWSTT-RAGT in addition to BWSTT alone may be more effective than BWSTT alone in improving dynamic balance, speed and endurance during gait [43], but the actual changes in gait pattern in kinetics are not yet cleared [44]. Additionally, in a comparison of BSWTT-RAGT and Conventional Physiotherapy [45], the BSWTT-RAGT group showed a significant improvement between groups.

To summarise these results, some showed that BWSTT-RAGT was more effective than the target group, while others showed no significance. These results indicate that BWSTT-RAGT seems to be more effective than gait training with BWSTT alone, whether the robot or the therapist provides the assistance. Regarding gait assistance, it is unclear whether it is worthwhile to use the current exoskeletal robot. Nevertheless, from the viewpoint of dependency of neural plasticity and training dose, it is impractical for therapists to provide long-term assistance in gait rehabilitation, therefore the use of robots should be advantageous from a clinical point of view. In addition, the significance of combination therapy together with BWSTT-RAGT has been demonstrated to be more effective than conventional physiotherapy, indicating that exoskeleton robot-assisted training has potential, with further research expected in the future.

### Types of exoskeleton design

Amongst the selected studies, there were four BWSTT-type exoskeletal robots: Lokomat, Walkbot, RoboGait, and GEAR system. The GEAR system is a prototype of the Welwalk and is already in clinical use as of 2021. The features of the devices and the details of assistance methods and intervention protocols vary. The differences in gait exercise effects between them are also not yet clear [60].

Lokomat and Walkbot are characterised by restricted motion of the pelvic girdle in the sagittal plane. The pelvic girdle’s semi-fixation in a certain position may reduce the abnormal gait pattern of the lower-limb joints [61]. With Lokomat, the timing of each muscle’s activation during gait is changed by adjusting the speed and guidance force [61]. On the other hand, with Welwalk, the pelvis is not fixed by the device, and there is more freedom in the direction of movement as compared to that with Lokomat and Walkbot. Other than the feature of BWS on the treadmill, the Welwalk can be used in situations closer to overground walking.

Regarding the protocol for adjusting the assist, there are a wide variety of possible assist trajectories, assist volumes, torque values at the joint, and control strategies. All included studies employed assist-as-needed approaches tailored to the gait of individual patients [35–45]. In all studies reviewed, these settings were applied and adapted to the subjects’ gait using an exploratory and experimental approach. For comparisons of effectiveness, the details of the settings of these elements need to be considered.

### Summary of future research questions

Based on the results of this review, future research questions and directions are discussed. Firstly, are there purpose-specific combinations of exoskeleton-assisted BWSTT and effective combination therapies for use in patients with chronic hemiplegia? If there are, what are these purpose-specific combinations? Secondly, do the effects of various robotic devices on gait training differ among patients with chronic hemiplegia?

Currently, the clinical use of exoskeleton-assisted BWSTT in patients with chronic stroke remains unclear due to a lack of evidence. Large RCTs in which patient recruitment, numerical assisted adjustments, treadmill speed, and details of intervention protocols that are compared with a control group will be needed in the future. This may aid in determining the appropriate applications of exoskeleton-assisted BWSTT.

## Limitations

The quality of evidence has not been assessed in the literature. A greater range of intervention methodologies and non-specific selection of case types need to be included. The type and severity of subjects’ disability, as well as intervention methodologies and protocols, are not considered and included. To address these limitations in the future, a high-quality systematic review with an expanded scope is necessary to be conducted.

## Conclusions

This review suggests that exoskeletal robot-assisted BWSTT for patients with chronic stroke may be effective in improving walking function as 10 out of 11 studies showed the effectiveness of exoskeleton robot-assisted BWSTT in terms of outcomes contributing to improved gait function. However, the potential may be “to assist” and not because of using the robot. In other words, the effect could be attributed to assisting, irrespective of whether it is due to a robot or therapist.

Further studies are required to verify the effectiveness of BWSTT exoskeletal robotic training in patients with chronic stroke, strengthen the evidence on intervention protocols, and provide detailed information regarding the application of different robot types to enable best practise for the benefit of patients.

## Data Availability

Not applicable.

## Abbreviations

BBS: Berg balance scale
BWS: Body weight support
BWSTT: Body Weight-Supported Treadmill Training
BWSTT-RAGT: BWSTT-robot assisted gait training
BWSTT-TAGT: BWSTT-therapist assisted gait training
EEG: Electroencephalogram
FAC: Functional ambulation categories
FES: Functional electrical stimulation
GEAR: Gait exercise assist robot
10MWT: 10 Metre walk test
RCTs: Randomised controlled trials
tDCS: Transcranial direct current stimulation
VR: Virtual reality
